# Barriers and Facilitators to Participating in an Exercise Referral Scheme among Women Living in a Low Socioeconomic Area in Australia: A Qualitative Investigation Using the COM-B and Theoretical Domains Framework

**DOI:** 10.3390/ijerph191912312

**Published:** 2022-09-28

**Authors:** Grace McKeon, Chiara Mastrogiovanni, Megan Teychenne, Simon Rosenbaum

**Affiliations:** 1 Discipline of Psychiatry and Mental Health, University of New South Wales, Sydney, NSW 2052, Australia; 2 School of Population Health, University of New South Wales, Sydney, NSW 2052, Australia; 3 Institute for Physical Activity and Nutrition (IPAN), School of Exercise and Nutrition Sciences, Deakin University, Geelong, VIC 3217, Australia

**Keywords:** physical activity, exercise referral schemes, women, socioeconomic

## Abstract

Introduction: Despite the health benefits of regular physical activity, women experiencing socioeconomic disadvantage are at high risk of inactivity. Reasons are multifactorial but likely include broad structural and contextual factors, e.g., lack of access to physical activity programs, as well as individual and interpersonal factors, e.g., lack of motivation and childcaring responsibilities. Few studies among women of low socioeconomic position (SEP) have explored these factors in-depth, yet an understanding of these factors can help inform the development and improve the uptake of exercise referral schemes. The Theoretical Domains Framework (TDF) and COM-B model (capability, opportunity, motivation and behaviour) were employed to understand behaviours for intervention development. Therefore, using these behaviour change models, this study aimed to explore the barriers and facilitators influencing the use of an exercise referral scheme among women living in a socioeconomically disadvantaged area. Methods: Semi-structured interviews were conducted with women who had registered with a free exercise referral scheme (Live Life Get Active) and living in a low socioeconomic neighbourhood in Sydney, Australia. Reflexive thematic analysis and framework analysis were used to allow naturally identified themes to be determined and then allocated to theoretically driven domains. Results: Nine women were interviewed (aged 30–69 years). Eighteen themes were identified and mapped directly on to the six COM-B constructs. The most reported barriers to using the physical activity referral scheme related to the *opportunity* construct of the COM-B model, specifically childcare responsibilities, work commitments and environmental barriers. Key facilitators were enjoyment (*motivation*), no cost (*opportunity*), instructor led (*opportunity*) and social support (*opportunity*). Conclusion: Future exercise referral schemes targeting women living in low-SEP neighbourhoods should ensure programs are designed and delivered to overcome barriers aligned with the constructs of the COM-B model, particularly *opportunity*-related constructors.

## 1. Introduction

The physical and mental health benefits associated with regular physical activity are well established [1]. However, around 28% of the population are physically inactive [2], i.e., not meeting the World Health Organization guidelines of 150 min of moderate to vigorous activity per week [3]. Physical inactivity has been identified as the fourth leading risk factor for global mortality, causing an estimated 3.2 million deaths per year [4]. It is one of the leading contributors to the global burden of disease including noncommunicable disease mortality [5,6].

Increasing physical activity participation is a complex problem requiring a broader focus than simply individual level interventions [7,8]. For example, socioeconomic position (SEP) is a term used to describe an individual’s affluence or social standing, referencing factors such as education, income levels and occupation [9]. Mental health is socioeconomically patterned whereby low SEP populations are most at risk of mental ill-health [10]. There are four domains of physical activity including occupational, domestic, transportation and leisure-time activity [11]. SEP is a strong predictor of participation in the leisure-time domain, i.e., physical activity performed during free time such as walking, strength-training or dancing [12,13]. This domain of activity is also strongly associated with mental health [14]. Evidence suggests population physical activity levels follow a social gradient, whereby those who are more advantaged (e.g., those of higher SEP with higher education levels, higher incomes, living in higher socioeconomic neighbourhoods) are more likely to be physically active in this domain, compared to those who are less advantaged [12,15,16].

The reasons are likely complex, entrenched in broad structural and contextual factors, as well as in individual and interpersonal factors [17]. Studies examining individual influences on physical activity have identified that lack of financial resources, lack of transport, illness, disability, and coping styles help explain socioeconomic disparities in physical activity levels [18]. Importantly, the risk of physical inactivity is high among people living in a socioeconomically disadvantaged area, independent of one’s own individual socioeconomic circumstances [9,19]. Physical, interpersonal and environmental factors influencing activity levels amongst people in low SEP neighbourhoods include perceptions of access and safety, e.g., to exercise in the local park [20,21], social support and neighbourhood social cohesion [22].

Further inequalities in physical activity participation are associated with gender, where in almost all countries women are less active than men [2,23]. The difference in physical activity levels between men and women is even greater in high- and middle-income countries with higher income inequality compared to low-income countries [24]. Barriers to physical activity for women are multifactorial but include socially determined resources such as time and income [25] and childcaring responsibilities [26]. Women living in socioeconomically disadvantaged areas therefore experience intersectional disadvantage and engage in less leisure-time physical activity compared to those from high socioeconomic areas [9,27]. Subsequently, women are at higher risk of experiencing co-morbid health conditions that are related to physical inactivity such as heart disease, type 2 diabetes, depression and anxiety [28,29]. Women from socioeconomically disadvantaged areas are therefore an important group to target for physical activity promotion and health interventions should be designed to directly address barriers to physical activity associated with social disadvantage [30].

Exercise referral schemes are used internationally to promote physical activity participation [31]. Schemes must, however, consider the context, and barriers and facilitators to engagement, specifically in disadvantaged populations [32]. An understanding of how and why existing schemes work or do not work will help to inform future efforts [33]. Yet, currently, little research exists that comprehensively explores factors within evidence-based frameworks for behaviour change. Pertinent frameworks of behaviour change suggest that individuals need to have the *capability, opportunity* and *motivation* to be able to make changes [34]. The COM-B model of behaviour [35] has been widely used in physical activity research to identify what needs to change in order for behaviour change to be effective [36,37]. It is based on the notion that behaviour change will only occur if an individual possesses the *capability* to enact the behaviour, the *opportunity* to enable the behaviour and have the *motivation* to perform the behaviour while overcoming the automatic *motivation* to not engage or to engage with another competing behaviour. The Theoretical Domains Framework (TDF) is an evidence-based and validated approach used in conjunction with the COM-B, building on the systems identified in the COM-B to further identify the underlying barriers and facilitators to behaviour change [38,39]. Some examples of domains the framework explores are knowledge (about the behaviour), beliefs about capabilities, emotions, and social influences. This can be used to inform the development of evidence-based interventions and facilitate implementation of those interventions.

This study therefore aimed to examine the barriers and facilitators to using an existing free exercise referral scheme among women living in a socioeconomically disadvantaged area in Sydney, Australia.

## 2. Methods

### 2.1. Study Design

This is a qualitative, descriptive study [40] informed by pragmatic research paradigm [41] and reported based on the standards for reporting qualitative research (SRQR) to ensure methodological rigour and transparency (see Supplementary Materials) [42]. Individual interviews with thematic analysis [43,44] was the most suitable approach to understand the opinions of women surrounding their personal barriers and facilitators to utilising a free exercise referral scheme in a real world setting. Researcher reflexivity is acknowledged to influence results due to researcher beliefs, experiences and context.

### 2.2. Participants

Participants were recruited through Live Life Get Active (LLGA) https://livelifegetactive.com/, an Australian health promotion charity providing free outdoor group exercise classes across over 100 locations. Members need to have registered online before attending exercise classes and can be referred by a health professional for chronic condition management or self-refer. LLGA work with government, primary health networks, commercial organisations, charities and council partners to fund the free programs. LLGA has a camp at Lang Park in St Mary’s. Personal trainers run five classes per week, including boxing, yoga and cross training.

An advertisement was sent out by LLGA via email to people who had registered with LLGA at the St Mary’s site in Sydney Australia. In Australia, socioeconomic disadvantage is assessed by geographic area using a score called the Socio-Economic Index for Areas (SEIFA), which is based on attributes including income, educational attainment and unemployment [45]. St Mary’s, is a suburb in Western Sydney that has scored in the lowest 10% of suburbs on the ABS SEIFA [45,46]. Participants also needed to meet the following criteria:

1. Female;
2. Aged 18 years or older;
3. Registered with LLGA’s St Mary’s camp (Lang Park, St Mary’s, NSW, Australia) and either attended or not attended sessions;
4. Living in St Mary’s.

Ethics approval was gained from UNSW Human Research Ethics Committee prior to recruitment (HC210645).

### 2.3. Procedures

Twenty-five women contacted the research team and registered their interest in taking part. All women were contacted and the eleven who responded were screened. Nine were eligible, consented to participate and followed through with interviews. Eligible participants took part in semi-structured one-on-one interviews for approximately 30–40 min via video call. The interviews were conducted by a member of the research team, with expertise in physical activity promotion and qualitative research (GM). The COM-B model and Theoretical Domains Framework (TDF) were used to develop the interview schedule. Interview questions related to participants’ understanding of the role of physical activity and its benefits, consequences of inactivity, the physical activity guidelines, perceived barriers and facilitators to physical activity participation including the LLGA exercise referral scheme [35]. The full interview guide is in the Supplementary Material. Interviews were transcribed verbatim and modified to remove identifying information. Transcripts were uploaded to NVivo12 (QSR International, Melbourne, Australia) qualitative analysis software.

### 2.4. Analysis

Both reflexive thematic analysis [43] and framework analysis [35] were used to allow naturally identified themes to be determined and then allocated to pre-selected theoretically driven domains to assist in identifying the barriers and facilitators to using a free exercise referral scheme. Following Braun and Clarkes’ [43] steps of undertaking reflexive thematic analysis, firstly, two researchers (GM and CM) became familiar with the interviews after multiple readings of the transcripts (Step 1 (familiarisation)). This included note taking to help facilitate immersion in data. Secondly, GM and CM subsequently independently coded each interview (Step 2 (Generating codes)). An inductive thematic analysis was conducted to identify new “candidate” themes by combining similar codes to create major categories using a thematic map [43] (Step 3 (Constructing themes)). These themes were then deductively mapped against the TDF domains and COM-B. The deductive mapping of themes was conducted by GM and CM. Any disagreements were discussed with MT. These themes were then reviewed, checked against the data set and “candidate” theme names were provided, clearly reflecting the meaning of each (Steps 4 and 5 (Revising and defining themes)). Finally, the results were written up, which enabled themes to continue to be tested (and refined if needed as a final stage of analysis) as to how well they answered the research question. Quotes were anonymised and presented to illustrate the core meaning of themes (Step 6 (Producing the report)).

### 2.5. Reflexivity

GM (PhD) undertook the qualitative interviews with participants. GM is a female research fellow, with previous experience in qualitative research in physical activity and mental health. This may have influenced the questions that the interviewer delved further into, for example when participants spoke about the benefits of physical activity on their mood. Researchers involved in the analysis (GM and CM) are accredited exercise physiologists meaning they have brought additional knowledge to the interpretation of the data. Both GM and CM do not live in low SEP areas. While this may have affected the interpretation of the results, the themes were deductively mapped to pre-established domains. All remaining authors were not involved in the qualitative interviews and analysis of the data; however, they were given the opportunity to review themes and suggest different interpretations of the data.

We also acknowledge there is the potential for social desirability bias given the participants were recruited through LLGA. However, participants were assured that the research study was being conducted independently of LLGA and the no identifying information (people or organisations) would be published/presented. We also reinforced at the beginning of each interview that researchers were only interested in their thoughts and perspectives on physical activity and referral pathways, and that there were no right or wrong answers.

## 3. Results

### 3.1. Participant Characteristics

Nine women living in St Mary’s, Sydney, participated in an interview between November and December 2021. The age of participants ranged from 30 to 69 years with a mean age of 53 years (SD = 12.9). All women had completed high school at least at year-ten level; four had a TAFE certificate or diploma and two had completed a bachelor or grade certificate at university. Two women were living alone, one lived with a partner and the others were living with children, either with or without a partner. Two were unemployed, four retired and three currently working. All but one of the women had attended LLGAs in person exercise sessions; however, due to COVID-19 restrictions, many had not attended within the past six months of completing the interview. Interviews ranged from 26 to 49 min and were on average 35 min (SD = 8.23). Participants are referred to throughout the manuscript using pseudo names.

### 3.2. TDF and COM-B Analysis: Barriers and Facilitators to Using a Free Exercise Referral Scheme

**Figure 1:**
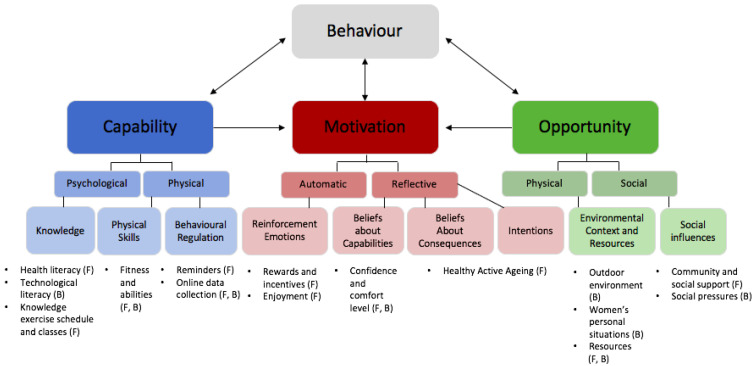
COM-B Framework, corresponding TDF domains and theme structure. The COM-B Framework component of this figure was adapted from McDonagh L et al. 2018 [47]. “B” represents a barrier to group physical activity and “F” represents a facilitator.

The women in this study identified several barriers and enablers to using a free, group based physical activity referral scheme. A total of eighteen themes were constructed, mapped to nine TDF domains and the six COM-B components relating to *capability*, *opportunity* and *motivation* (Figure 1). Participant quotes classified according to the theme structure are shown in the Supplementary Material.

### 3.3. Capability

#### 3.3.1. Psychological Capability—Knowledge

1. Health Literacy

All participants identified or partly identified the physical activity guidelines correctly. Regardless of whether participants were meeting these guidelines themselves, all discussed the health benefits associated with participating in physical activity including improvements in mood, weight, physical function, and heart health.

2. Technological Literacy

Being technologically illiterate was identified as a barrier to the referral scheme given the only registration option was online. Technological literary describes the familiarity with access and use of digital information and devices. While most of those interviewed were confident in their abilities to use technology and did not have issues navigating online services that related to exercise classes, a few spoke about friends and family who are not technologically literate and how this is a barrier for them to participate.

“I have a friend who doesn’t even have a mobile phone. So that would definitely be a barrier for them.”

—Elsie, 60

3. Knowledge Exercise Schedule and Classes

Lack of knowledge about what the different types of exercise classes being offered by the exercise scheme (e.g., boxing, Pilates, cross-training) would involve was discussed as a barrier to participation for a couple of participants. A lot of participants discussed feeling nervous and lacking confidence to attend, particularly in their first few sessions.

“I don’t even understand what some classes would entail like boxing, I think it was Box Fit. I’ve got no idea if that’s full on.”

—Jada, 50

#### 3.3.2. Physical Capability—Physical Skills

1. Fitness and Abilities

Those who had not attended a session or who were reflecting back on when they were new to LLGA often had low self-efficacy in their ability to take part due to their current fitness or ability levels. Linking to ‘knowledge about types of exercises/classes’, some participants recommended that programs should contain detailed information to help participants choose the classes that best suited their abilities and that a beginner’s class would be a helpful option for new members that would encourage attendance. Among those that attended regularly, the varying levels of ability and ages among participants in LLGA sessions was seen as a facilitator.

“There are people of all different fitness levels, all different ages, and we all support each other to go further, within our journey.”

—Jenny, 30

To increase attendance, it was suggested to include “an easier class for people who do have trouble getting up and getting down again.”

—Mary, 69

#### 3.3.3. Physical Capability—Behavioural Regulation

1. Reminders

Participants reported that they would often forget to attend sessions due to other commitments. A facilitator to physical activity commonly discussed were reminders to help improve accountability. Most participants suggested that an automated text message reminder before classes or after a missed class would be helpful.

“I think a text message is a very good idea because sometimes you forget.”

—Camila, 61

2. Online Data Collection

The use of online data collection profiles to track progress was described as both a barrier and facilitator to physical activity. Most participants liked being able to reflect on their progress, including the opportunity to celebrate milestones such as attendance, but others were hesitant to use the online health tracking, in particular their weight, as they felt it was more important to focus on feeling fitter and stronger rather than having weight-focused goals. Overall, these were the individuals who preferred to provide less information online.

“I don’t like that either personally, always asking about your weight and if you’ve lost weight or if you’ve gained weight… I don’t want to be weighing myself all the time just to put that in, I mean because I don’t want to become obsessed with that.”

—Camila, 61

“I think [the online data collection] is helpful and the trainers will acknowledge when people have been there for significant sessions.”

—Elsie, 60

### 3.4. Opportunity

#### 3.4.1. Physical Opportunity—Environmental Context and Resources

1. Outdoor Environment

The weather was discussed as a barrier to participation, with a few of participants saying that rain and excessive heat prevented them from attending classes. A lack of shelter or suitable alternatives to the public park during these weather events was reported as a barrier.

“In the morning most times are okay, but you know if you get those really hot days it’s a little bit hot. So you know that’s the downside of being outdoors. When it rains obviously it’s a bit of an issue as well.”

—Mahlia, 67

The role of the built environment was discussed as a factor influencing participation. Some reported that they enjoy exercising outdoors in an environment with green space and that recent building developments in the area reduce the motivation to be active. Concerns about safety while training in a public park were also perceived as a barrier due to the presence of members of the public, the lack of security for storing possessions and environmental hazards.

“There used to be like people sort of drug users walking past. There was sort of undesirable people which would sort of walk past, and you have your belongings on the ground. I used to think I hope no one picks up my bag and runs off with it.”

—Susan, 42

“I have to say that it does because I used to quite enjoy but now they all got bulldozed for houses and we’re going to have a massive housing development. And I like greenery. So you go on a walk, I want to walk where there’s trees and greenery and birds, and all that sort of stuff.”

—Jada, 50

2. Women’s Personal Situations

Women’s personal situations which affected physical activity levels included finances, working schedule and family or childcare responsibilities. The fact that the exercise referral scheme program was free was one of the most frequently reported facilitators discussed by almost all. Women repeated that without free community classes, they would not otherwise be able to access any other exercise services.

“Truthfully, the fact that it was free. That was a big plus for me because, like I had stopped working and one of the reasons why I had, is you know I needed to help my daughter a little bit more with her child. So, I’ve found there’s people like me in those classes and so, you know, now I have to really watch money. I don’t know if it’s a good thing or not but certainly that was a big factor for me.”

—Camila, 61

“I used to be involved in a gym but I’m on limited income so you know some gym memberships can get a little bit expensive”

—Mahlia, 67

Family responsibilities often impeded participation in exercise for women, and was even noted by participants who did not have children living at home, with those women reflecting on when they had young children at home and found it difficult to exercise with family responsibilities.

“…you know you’re tired, you’re raising a family. You’re working.”

—Camila, 61

These women supported other women bringing children to exercise classes and discussed the support they would have like to receive when they had young children, such as childcare during exercise classes.

“… last year, we had a fantastic opportunity where a lady‘s son who was probably about eight, he exercised with us, and the trainer was so beautiful…. He said to the child hey, you can be my supervisor, you can make sure all the ladies are doing the exercise, and that it boosted the kids confidence so not just people without children, but people with children can feel comfortable there….”

—Jenny, 30

3. Resources

Resources including childcare, exercise trainers and time were both barriers and enablers to using the physical activity referral scheme. Women who were currently working reported a lack of time to exercise, while all of those who were retired did not. Friendly and welcoming trainers with the skills to accommodate all abilities of exercisers in the classes were a facilitator to physical activity reported by all participants. However, a suggestion to increase inclusion and reach was to have trainers with representation from diverse communities. Overall, the most helpful resources varied depending on women’s personal situations but having good trainers and a range of time options for classes were the most helpful resources currently in place, with childcare a highly suggested resource to add for exercise class providers, although many may not have the ability to implement it.

“If the places have trainers from my community too. Someone knows them and then families and that go and support each other.”

—Layla, 53

“If they have another program for the kids while the mums are doing the exercising the kids can too.”

—Tess, 43

#### 3.4.2. Social Opportunity—Social Support and Social Influences

Themes related to social opportunity included (i) community, social support, influence and interactions as well as (ii) social status, pressures and comparisons.

1. Community and Social Support

Community and social support were seen as enablers to physical activity and a couple of participants spoke about meeting friends through community exercise classes and going for coffees after classes. Participants said that they were more comfortable attending their first group session if they had a friend or family member attend with them. Additionally, many stated that having family members or friends who are active themselves was motivating for their own activity.

“I’d sort of told a couple of my other friends about it so like the three of us sort of like, you know, went together that first week, so you know like I knew somebody.”

—Mahlia, 67

“You become friends with people and you tend to after training, you get to sit for five minutes and just chat and it might turn into, hey, what do you doing? Want to meet up at Woolworths and continue shopping together and get groceries?”

—Tess, 43

2. Social Pressures

Social status, pressures and comparisons were regularly discussed as barriers to using other exercise referral schemes including commercial gyms. Participants reported that attending gyms often made them feel uncomfortable or embarrassed because they did not own the right clothing or know how to use the equipment.

“Society has really created this persona that you have to look, act and afford to be able to be active…..Every time I wanted to go there [to a gym], I felt like I had to wear expensive clothing.”

—Jenny, 30

“I have looked at going to gyms in the past, but I look at the machines and I don’t even know how to use them.”

—Jada, 50

The community exercise program was described as a facilitator to help overcome pressures related to social status.

“My mental health didn’t feel comfortable being in a gym because of my social status, not having much money, and so, when I walked past Live Life Get Active I was like, they told me it was free they told me what they run, and I was just intrigued, because I since childhood has been a community person.”

—Jenny, 30

### 3.5. Motivation

#### 3.5.1. Automatic Motivation—Reinforcement Emotions

1. Rewards and Incentives

It was discussed that challenges and competitions were facilitators to physical activity adherence. External rewards such as gift vouchers or movie tickets were not considered as important as intrinsic rewards such as improving mental health and fitness.

“Every now and then maybe having some sort of competition. Sign up a friend or something.”

—Elsie, 60

2. Enjoyment

Participants spoke of their motivation to attend exercise sessions to be mainly due to enjoyment. This was an important facilitator and often a point raised as to why most participants attend these classes over engaging in other physical activity options such as attending a gym. The enjoyment factor was frequently linked to the variety of class options and the social component.

“I do like going to a class, yeah I personally it’s not so much the social thing really, but yeah I do I do just love the structure of the class I like the instructor up there, the facilitator what do you call it and yeah, it is a bit social, that’s kind of a bonus but I just like the whole thing of a class.”

—Camila, 61

#### 3.5.2. Reflective Motivation—Beliefs about Capabilities

1. Confidence and Comfort Level

Increased self-efficacy was a facilitator to attending group exercise classes and most participants reported that the more classes they attended, the more confident they felt continuing to attend with their first few sessions being the hardest to show up to. Some participants reported that attending their first sessions with a friend or family member helped increase their initial confidence and comfort levels. A lack of confidence often related to a lack of knowledge about exercise class requirements and/or feeling too unfit to participate in exercise classes.

“You’re able to get this confidence boost about you, you’re able to validate and feel proud of yourself.”

—Jenny, 30

“I was attending some other classes, they were for over 50 s, or over 55 s and because I wasn’t all that confident about joining a regular open aged class.”

—Mary, 69

#### 3.5.3. Reflective Motivation—Beliefs about Consequences and Intentions

1. Healthy Active Ageing

The most common physical activity goals that motivated participants to use the referral scheme related to weight loss, maintaining independence and preventing age related health decline. However, many reported that their initial goal changed they attended exercise sessions, with many saying they continue to attend primarily for mental health benefits. Knowing the wide range of benefits of exercise was a motivating factor for participants to exercise and allowed participants to recognise goals that exercise could help them with.

“If you don’t use it, you lose it, so it’s about health, staying flexible, getting less aches and pains, sort of wards off illness.”

—Elsie, 60

“Weight loss is a bonus but it’s also getting to meet people, but also it’s something that I can do for me with, and, you know, like, and it does help mentally for me. It is a mental thing because I do have those mental challenges so it’s that 45 min of not thinking of all the other stuff and just focusing on me.”

—Tess, 43

## 4. Discussion

The purpose of this study was to explore the barriers and facilitators to using an exercise referral scheme among women living in a low socioeconomic area in Australia. The use of the COM-B model provided an overview of factors contributing to participation in the referral scheme and mapping the data onto nine TDF domains ensured a detailed analysis of the determinants of behaviours. The findings are important to consider when designing exercise referral schemes.

The most prominent barriers to using the exercise referral scheme were associated with the *opportunity* construct. Core themes attributed to physical opportunity included financial barriers, work and family commitments, childcare responsibilities and environmental barriers. Many women discussed experiencing financial stress and the cost of other exercise referral schemes including gym memberships as prohibitive. The no cost associated with the LLGA sessions was a key facilitator for using the scheme. Women also discussed the impact of family commitments on their ability to use the referral scheme regularly. This finding is unsurprising given women take on the majority of the worlds unpaid care work compared to men [48,49]. Previous research has shown that as men’s employment hours increase, women’s family work hours increase and subsequently their physical activity levels decrease [25]. The women interviewed were however supportive of children being able to attend classes with their mothers and suggested that this option be more publicised. Social barriers to participation included concerns of judgement over social status, appearance and lack of suitable clothing from peers. Previous research has shown that these barriers are particularly pertinent among women than men, and even more so among women with poor body satisfaction [50].

Other major barriers mapped to the *opportunity* construct were related to the environment. Many reported perceived safety concerns in the local neighbourhood where the exercise sessions were held, including fears their belongings would be stolen if left unsupervised. This aligns with previous research showing that perceived safety affects physical activity participation in local public spaces, particularly among women [51]. Other barriers to attending were extreme weather events, although notably the interviews were conducted during the peak of Australian Summer. While the LLGA sessions are conducted in a local park, a lack of green space and increasing developments in the area were mentioned as barriers to being active. Greener neighbourhoods are often associated with improved mental health and levels of physical activity [52], which further emphasises the importance of urban planners ensuring greater access to quality green space in socioeconomically disadvantaged neighbourhoods. These *opportunity* related barriers which were raised are closely linked to socioeconomic position, and although our research did not compare barriers and facilitators to those in high socioeconomic areas, previous research has shown that many of these are specific to this group [18].

Overall, participants demonstrated the *capability* to be physically active. All participants showed good knowledge of the physical activity guidelines and the benefits of being active. Regarding physical capability, concerns about not being fit enough were felt by participants prior to attending their first session, however these feeling subsided once they had attended. This was predominantly because sessions were supervised by fitness instructors who provided motivation and were able to tailor exercises to all levels and abilities. Similarly to previous research [53], supervision by a trainer was a highly valued component of the referral scheme. To support women to feel confident to attend the first session, it is recommended that referral schemes have inclusive pathways and that referrers have a basic understanding of the types of exercise sessions offered and the level of support the program will provide. While capability of using the scheme was high, a recommendation to further support regular attendance was text message reminders, which have been shown to be an efficacious strategy/tool (in conjunction with planning and cash incentives) to increase physical activity (specifically weekly gym visits) amongst adults in the USA [54].

Themes associated with the *motivation* construct of the COM-B model revealed that enjoyment was a key facilitator to using the referral scheme. Participants were also motivated by the health benefits and social support gained through the group program. This relates to the findings in other studies showing that having someone to exercise with would increase women’s motivation to engage in the activity when they are referred [55]. For those who reported feeling apprehensive to first try the exercise referral scheme, social support from a friend or family member was discussed as a facilitator. Interestingly, many of these factors including enjoyment and social support are recommended when prescribing physical activity for mental health [14]. These factors may therefore mediate the relationship between physical activity and mental health, which is significant given women living in low socioeconomic areas are a particularly high-risk population for mental health problems [56]. There did not appear to be any major barriers related to motivation; however, notably, nine out of ten participants had adhered to at least one session so were likely already motivated.

### Limitations and Strengths

Firstly, we note the sampling bias inherent in the present study as a limitation given participants comprise of a selective group of individuals who had nearly all attended at least one LLGA exercise session. Future research should consider comparing the barriers and facilitators among adherers versus non-adherers to exercise referral schemes. In addition, the participants were predominantly over the age of 50, tertiary educated and were only recruited through one exercise referral scheme (LLGA) in one specific area of Sydney, Australia. Therefore, transferability of findings to may be limited. Lastly, to further improve the research quality, interviewee transcript review should be undertaken in future research studies [57].

A strength of the study is the qualitative design, enabling rich data and insights which would not have been possible using other study designs. In addition, the research was underpinned by a widely used and contemporary theoretic framework which allowed us to rigorously test and explore constructs. Lastly, the research targets an important population of women from low SEP who are a generally hard to reach group, at high risk of physical inactivity.

## 5. Conclusions

The present study took a novel approach adopting both the COM-B model and TDF to identify factors that influence adherence to a physical activity referral scheme among women living in a low socioeconomic area. Although barriers and facilitators identified encompassed all six constructs of the COM-B model, physical and social *opportunity* barriers to participation were the most prominent. Our results suggest that exercise referrals to programs that are social, instructor led, enjoyable, inclusive of all abilities and free may help improve adherence to referral schemes amongst this population.

## Data Availability

Data available on request due to privacy restrictions. The data presented in this study are available on request from the corresponding author. The data are not publicly available due to privacy restrictions.

## Supplementary Materials

The following supporting information can be downloaded at: https://www.mdpi.com/article/10.3390/ijerph191912312/s1, Part S1: Interview Schedule; Part S2: SQRQ checklist; Part S3: Participant quotes classified according to the theme structure.
