# Supplementary material for: Barriers and Facilitators to Participating in an Exercise Referral Scheme among Women Living in a Low Socioeconomic Area in Australia: A Qualitative Investigation Using the COM-B and Theoretical Domains Framework
Source: Int J Environ Res Public Health. 2022 Sep 28;19(19):12312. doi: 10.3390/ijerph191912312 (PMC9565931; doi:10.3390/ijerph191912312)
Supplement: Supplementary file 1 [file ijerph-19-12312-s001.zip › ijerph-1897397-supplementary.pdf]

Part S1. Interview Schedule

Group 1) Registered with LLGA but not attended

Capability:

1. What is your current understanding about the role of physical activity and what does it mean to you? (Prompt: health, fitness, social)
2. Are there any benefits you see/know of participating in PA/exercise? If yes, then what are they?
3. Are there any consequences that you see/know of not being active/exercising? If yes, what are they?
4. Do you know how much activity is recommended per week? If yes, what is it?
5. Do you feel you have the confidence to exercise on your own? Why/Why not? (Prompt: Feel self-conscious; too shy; don't like being in groups; prefer to exercise alone (or am I jumping ahead here?)
6. How about in a group? Which do you prefer?
7. What is the role of free community group classes like Live Life Get Active?

Motivation/Opportunities:

1. Over the past month, have you engaged in any physical activity and if what type and how often? And for what purpose? E.g., walking for transport, gardening, exercise? COVID?
2. Do your friends and/or family influence your physical activity? How? *(prompts: look after kids while you exercise; exercise-buddy; social media pressure; walking groups)*
3. How did you find out about Live Life Get Active and what motivated you to register?
4. What barriers have you faced to attending a LLGA session? If so, what are they (prompts: lack time, motivation, lack childcare, transport etc)
5. Are there any other barriers that you think would prevent you from attending group community classes like this one?
6. Is there any support or resources you think would help overcome these barriers? If yes, what are these? *(prompts: transport cards, access to creche' (or kids join?), different hours of program delivery, text message prompts or online LLGA community for social support/motivation/accountability etc)*

Group 2) Registered with LLGA and attended at least 1 session

Capability:

1. What is your current understanding about the role of physical activity and what does it mean to you? (Prompt: health, fitness, social)
2. Are there any benefits you see/know of participating in PA/exercise? If yes, then what are they?
3. Are there any consequences that you see/know of not being active/exercising? If yes, what are they?
4. Do you know how much activity is recommended per week? If yes, what is it?
5. Do you feel you have the confidence to exercise on your own? Why/Why not? (Prompt: Feel self-conscious; too shy; don't like being in groups
6. How about in a group? Which do you prefer?
7. What is the role of free community group classes like Live Life Get Active?

Motivation/Opportunities:

1. Over the past month, have you engaged in any physical activity and if what type and how often? And for what purpose? E.g. walking for transport, gardening, exercise? COVID?
2. Do your friends and/or family influence your physical activity? How? *(prompts: look after kids while you exercise; exercise-buddy; social media pressure; walking groups)*
3. How did you find out about Live Life Get Active and what motivated you to register and attend?
4. Are there any other factors that have helped facilitate your engagement in the LLGA program? If yes, what are they? (Prompts: time of delivery, cost-free, close to home, hubby can look after kids at that time etc)
5. Have you faced any barriers in attending the LLGA program? If so, what are they (prompts: lack time, motivation, lack childcare, transport etc)
6. Are there any support or resources you think would help overcome these barriers? If yes, what are these? *(prompts: transport cards, access to creche' (or kids join?), different hours of program delivery, text message prompts or online LLGA community for social support/motivation/accountability etc)*
7. Is there anything in particular you have liked/disliked about the LLGA program so far? Please explain.
8. During the program, did you have any concerns about it? If yes, what were they? e.g., timing of the sessions, types of sessions etc.
9. Anything else you would like to add about the LLGA program?

Part S2. SQRQ checklist

Item Number	Item	Description
S1	Title	Barriers and facilitators to participating in a free exercise referral scheme among women living in a low socioeconomic area in Australia: A qualitative investigation using COM-B and Theoretical Domains Framework.
S2	Abstract	Introduction: Despite the health benefits of regular physical activity, women experiencing socioeconomic disadvantage are at high risk of inactivity. Reasons are multifactorial but likely include broad structural and contextual factors e.g., lack of access to physical activity programs, as well as individual and interpersonal factors e.g., lack of motivation and childcaring responsibilities. Few studies among women of low socio-economic position (SEP) have explored these factors in-depth, yet an understanding of these factors can help inform the development and improve the uptake of exercise referral schemes. The Theoretical Domains Framework (TDF) and COM-B model (capability, opportunity, motivation and behaviour) have been employed to understand behaviours for intervention development. Therefore, using these behaviour change models, this study aimed to explore the barriers and facilitators influencing the use of an exercise referral scheme among women living in a socioeconomically disadvantaged area.<br><br>Methods: Semi-structured interviews were conducted with women who had registered with a free exercise referral scheme (Live Life Get Active) and living in a low socio-economic neighbourhood in Sydney, Australia. Reflexive thematic analysis and framework analysis were used to allow naturally identified themes to be determined and then allocated to theoretically-driven domains.<br><br>Results: Nine women were interviewed (aged 30-69 years). Eighteen themes were identified, and mapped directly on to the six COM-B constructs. The most reported barriers to using the physical activity referral scheme related to the *opportunity* construct of the COM-B model, specifically childcare responsibilities, work commitments and environmental barriers. Key facilitators were enjoyment (*motivation*), no cost (*opportunity*), instructor led (*opportunity*) and social support (*opportunity*).<br><br>Conclusion: Future exercise referral schemes targeting women living in low SEP neighbourhoods should ensure

		programs are designed and delivered to overcome barriers aligned with the constructs of the COM-B model, particularly *opportunity* related constructors.
S3	Problem formulation	Women living in socio-economically disadvantaged areas therefore experience intersectional disadvantage and engage in less leisure-time physical activity compared to those from high socio-economic areas. Subsequently, women are at higher risk of experiencing co-morbid health conditions that are related to physical inactivity such as heart disease, type 2 diabetes, depression and anxiety. Women from socioeconomically disadvantaged areas are therefore an important group to target for physical activity promotion and health interventions should be designed to directly address barriers to physical activity associated with social disadvantage.
S4	Purpose or research question	This study therefore aimed to examine the barriers and facilitators to using an existing free exercise referral scheme among women living in a socio-economically disadvantaged area in Sydney, Australia.
S5	Qualitative approach and research paradigm	This is a qualitative, descriptive study informed by pragmatic research paradigm and reported based on the standards for reporting qualitative research (SRQR) to ensure methodological rigour and transparency. Individual interviews with thematic analysis was the most suitable approach to understand the opinions of women surrounding their personal barriers and facilitators to utilising a free exercise referral scheme in a real world setting. Researcher reflexivity is acknowledged to influence results due to researcher beliefs, experiences and context.
S6	Researcher characteristics and reflexivity	GM (PhD) undertook the qualitative interviews with participants. GM is a female research fellow, with previous experience in qualitative research in physical activity and mental health. This may have influenced the questions that the interviewer delved further into, for example when participants spoke about the benefits of physical activity on their mood. Researchers involved in the analysis (GM and CM) are accredited exercise physiologists meaning they have brought additional knowledge to the interpretation of the data. Both GM and CM do not live in low SEP areas. While this may have affected the interpretation of the results, the themes were deductively mapped to pre-established domains. All remaining authors were not involved in the qualitative interviews and analysis of the data however, they were given the opportunity to review themes and suggest different interpretations of the data.<br><br>We also acknowledge there is the potential for social desirability bias given the participants were recruited through LLGA. However, participants were assured that

		the research study was being conducted independently of LLGA and the no identifying information (people or organisations) would be published/presented. We also reinforced at the beginning of each interview, that researchers were only interested in their thoughts and perspectives on physical activity and referral pathways, and that there were no right or wrong answers.
S7	Context	Participants were recruited through Live Life Get Active (LLGA) https://livelifegetactive.com/, an Australian health promotion charity providing free outdoor group exercise classes across over 100 locations. Members need to have registered online before attending exercise classes and can be referred by a health professional for chronic condition management or self-refer. LLGA work with government, health networks, commercial organisations, charities and council partners to fund the free programs. LLGA has a camp at Lang Park in St Mary's. Personal trainers run five classes per week, including boxing, yoga and cross training.
S8	Sampling strategy	An advertisement was sent out by LLGA via email to people who had registered with LLGA at the St Mary's site in Sydney Australia.
S9	Ethical issues pertaining to human subjects	Ethics approval was gained from UNSW Human Research Ethics Committee prior to recruitment (HC210645).
S10	Data collection methods	Nine women living in St Mary's, Sydney, participated in an interview between November and December 2021.<br><br>Eligible participants took part in semi-structured one-on-one interviews for approximately 30-40 minutes via video call. The interviews were conducted by a member of the research team, with expertise in physical activity promotion and qualitative research (GM).
S11	Data collection instruments and technologies	The COM-B model and Theoretical Domains Framework (TDF) were used to develop the interview schedule. Interview questions related to participants' understanding of the role of physical activity and it's benefits, consequences of inactivity, the physical activity guidelines, perceived barriers and facilitators to physical activity participation including the LLGA exercise referral scheme. The full interview guide is in the Supplementary Material.
S12	Units of study	Twenty-five women contacted the research team and registered their interest in taking part. All of the women were contacted and the eleven who responded were screened. Nine were eligible, consented to participation and followed through with interviews.
S13	Data processing	Interviews were transcribed verbatim and modified to remove identifying information. Transcripts were

		uploaded to NVivo12 (QSR International, Melbourne, Australia) qualitative analysis software.
S14	Data analysis	Both reflexive thematic analysis and framework analysis were used to allow naturally identified themes to be determined and then allocated to pre-selected theoretically-driven domains to assist in identifying the barriers and facilitators to using a free exercise referral scheme. Following Braun and Clarkes steps of undertaking reflexive thematic analysis, firstly two researchers (GM and CM) firstly became familiar with the interviews after multiple readings of the transcripts (Step 1 (familiarisation)). This included note taking to help facilitate immersion in data. Secondly, GM and CM subsequently independently coded each interview (Step 2 (Generating codes)). An inductive thematic analysis was conducted to identify new themes (Step 3 (Constructing themes)). These themes were then deductively mapped against the TDF domains and COM-B. The deductive mapping of themes was conducted by GM and CM. Any disagreements were discussed with MT. These themes were then reviewed, checked against the data set and 'candidate' theme names were provided, clearly reflecting the meaning of each (Steps 4 and 5 (Revising and defining themes)). Finally, the results were written up, which enabled themes to continue to be tested (and refined if needed as a final stage of analysis) as to how well they answered the research question. Quotes were anonymised and presented to illustrate the core meaning of themes (Step 6 (Producing the report)).
S15	Techniques to enhance trustworthiness	Researcher reflexivity is acknowledged in the manuscript and how this may influence the results due to researcher beliefs, experiences and context.<br><br>We did not use participant checking as a technique and this has been added as a limitation.
S16	Synthesis and interpretation	The women in this study identified several barriers and enablers to using a free, group based physical activity referral scheme. A total of 18 themes were constructed, mapped to nine TDF domains and the six COM-B components relating to capability, opportunity and motivation. Please refer to Figure 1 for further detail.
S17	Links to empirical data	Please see supplementary material for exemplar quotes for each theme.
S18	Integration with prior work, implications, transferability, and contribution(s) to the field	The use of the COM-B model provided an overview of factors contributing to participation in the referral scheme and mapping the data onto nine TDF domains ensured a detailed analysis of the determinants of behaviours. The findings are important to consider when designing exercise referral schemes.

		The most prominent barriers to using the exercise referral scheme were associated with the *opportunity* construct. Core themes attributed to physical opportunity included financial barriers, work and family commitments, childcare responsibilities and environmental barriers. Women also discussed the impact of family commitments on their ability to use the referral scheme regularly. This finding is unsurprising given women take on the majority of the worlds unpaid care work compared to men. Previous research has shown that as men's employment hours increase, women's family work hours increase and subsequently their physical activity levels decrease. Social barriers to participation included concerns of judgement over social status, appearance and lack of suitable clothing from peers.<br><br>Other major barriers mapped to the *opportunity* construct were related to the environment. Many reported perceived safety concerns in the local neighbourhood where the exercise sessions were held, including fears their belongings would be stolen if left unsupervised. This aligns with previous research showing that perceived safety affects physical activity participation in local public spaces, particularly among women. Other barriers to attending were extreme weather events, although notably the interviews were conducted during the peak of Australian Summer. While the LLGA sessions are conducted in a local park, a lack of green space and increasing developments in the area was mentioned as a barrier to being active. Greener neighbourhoods are often associated with improved mental health and levels of physical activity, which further emphasises the importance of urban planners ensuring greater access to quality green space in socioeconomically disadvantaged neighbourhoods. These *opportunity* related barriers which were raised are closely linked to socio-economic position, and although our research did not compare barriers and facilitators to those in high socio-economic areas, previous research has shown that many of these are specific to this group.<br><br>Overall, participants demonstrated the *capability* to be physically active. All participants showed good knowledge of the physical activity guidelines and the benefits of being active. Similarly to previous research, supervision by a trainer was a highly valued component of the referral scheme. To support women to feel confident to attend the first session, it is recommended that referral schemes have inclusive pathways and that referrers have a basic understanding of the types of

		exercise sessions offered and the level of support the program will provide. While capability to using the scheme was high, a recommendation to further support regular attendance was text message reminders, which has been shown to be an efficacious strategy/tool (in conjunction with planning and cash incentives) to increase physical activity (specifically weekly gym visits) amongst adults in the U.S.<br><br>Themes associated with the *motivation* construct of the COM-B model revealed that enjoyment was a key facilitator to using the referral scheme. Participants were also motivated by the health benefits and social support gained through the group program. This relates to the findings in other studies showing that having someone to exercise with would increase women's motivation to engage in the activity when they are referred. Interestingly, many of these factors including enjoyment and social support are recommended when prescribing physical activity for mental health. These factors may therefore mediate the relationship between physical activity and mental health, which is significant given women living in low socio-economic areas are a particularly high-risk population for mental health problems.
S19	Limitations	The sampling bias inherent in the present study is a limitation given participants comprise of a selective group of individuals who had nearly all attended at least one LLGA exercise session. Future research should consider comparing the barriers and facilitators among adherers versus non-adherers to exercise referral schemes. In addition, the participants were predominantly over the age of 50, tertiary educated and were only recruited through one exercise referral scheme (LLGA) in one specific area of Sydney, Australia. Therefore, transferability of findings to may be limited. Lastly, to further improve the research quality, interviewee transcript review should be undertaken in future research studies.
S20	Conflicts of interest	The authors have no conflicts of interest to disclose.
S21	Funding	This work was funded by LLGA.

Part S3. Participant quotes classified according to the theme structure.

COM-B		TDF	Themes	Quotes
**Capability**	Psychological	Knowledge	Health literacy	*"Well, I suppose it depends what you're doing, like with walking I think they say at least five times a week for good half hour walk but other than that, other exercise, no I'm not really sure."*<br><br>*"They recommend like at least three to four per week or depending, but then, like, I mean, each to their own like everyone's different so we know we all have different bodies, different, you know, activities going on and that so and, but some people, they wouldn't really need to exercise but they just do it because you know they think they need to. I wish I was one of those."*<br><br>*"I think it's at least 30 minutes every day."*<br><br>*"I do know it's good for physical and mental health, weight loss, depression and all the stuff it helps with that."*<br><br>*"If you, if you don't use it, you lose it so it's about you know health, staying flexible, yeah, getting less aches and pains, you know, sort of wards off illness and helps you to lose weight, yeah and also good for the mood as well for mental health."*<br><br>*"Making yourself feel better mood wise, also losing weight, like maintaining weight. Also, for your, for your heart as well and the muscles and all that. Strengthening."*
			Technological literacy	*"It's all online like you got to register and computer and phone and all that. If I can get their head around that they're fine, or we might go on and show it to them."*<br><br>*"I have a friend who doesn't even have a mobile phone. So, you know. Yeah, so that would be definitely a barrier for them."*
			Knowledge about types of exercise/classes	*"I don't even understand what some classes would entail like boxing, I think it was Box Fit. I've got no idea if that's full on."*
	Physical	Physical skills	Fitness and abilities	*"Not only trying to help the young ones to be getting fit and you know healthy, but for our older generation because our older generation doesn't ever you know like they've only supported people that were in sports and doing sports stuff, but they've never had physical activities within them like yeah, and they love it, like the young trainers and that they love it when they see all the oldies."*<br><br>When asked if the classes cater for all different types of levels and abilities, *"I think so cause I think they give options you know you can have an easier option then you can perhaps have a little more advanced options."*<br><br>*"It's amazing for mental health, because you go there, and there are people of all different fitness levels, all different ages, and we all support each other to go further, within our journey."*<br><br>*"I think too it's just the trainer like if you've got particular health issues, and certain exercises don't suit. There's an alternative way of doing it, or you might not do this you might do something else, you know, so he's really good and I think that's a safer way of exercising like I think if you exercise by yourself sometimes there could be a danger of injury."*

				"There are some things I can't do but the instructor is really good and he has different options if you can't do something that's a bit more difficult. He says you know just do this instead and you don't feel you don't feel put down you just yeah well that's how it is I can't do that yet so I'll do this and that's."
		Behavioural regulation	Reminders	*"Text message reminders is fantastic. If we had that, because sometimes I'll be like doing something and I'm like, 'oh my god, five minutes to go, I've got to quickly go' and you know."*<br><br>*"I think a text message is a very good idea, because sometimes you forget and sometimes too like I go to look in the classes, look at the, you know they have like a roster of the classes and the times, but I quite often forget my password.''*<br><br>*"The trainers also have the option for you to message them so there's been a day where I just didn't want to feel like getting dressed, I've had a lot of bad news, I was going through some grievance, and I had the option to message my trainer and I said, 'look, you know, I'm probably either going to be late or probably won't show up.' And he said, 'you know what, it's okay if, if you don't feel that you're in the best position to come, that's alright. Just remember that there is online option if later on during the day you're feeling any better that you have that option, but either way, you know, just take care of yourself.'"*
			Online data collection	*"That's something I've really embraced and has encouraged me because me seeing how many sessions I've attended, I'm able to go wow, I've attended that many, I want to attend more because, oh wow, this person or he has reached their milestone of 50 sessions. I want to go because I want to reach the 50."*<br><br>*"I don't like that either personally, always asking about your weight and if you've lost weight or if you've gained weight… I don't want to be weighing myself all the time just to put that in, I mean because I don't want to become obsessed with that."*<br><br>*"I think [the online data collection] is helpful and the trainers will acknowledge when people have been there for significant sessions."*
**Opportunity**	Physical	Environmental context and resources	Environment and weather	*"Cause it's outdoors some people have said what about indoors and like if it's a rainy day or if it's like winter. Having it indoors so then not... and like then the kids are able to be secured."*<br><br>*"The fresh air, even though it is a bit hot in summer. I think being out in fresh air is a big factor."*<br><br>*"I guess Summer is a little bit hard you know like when it is it like nine thirty in the morning most times is okay but you know if you get those really hot days it is a bit, little bit hot. So, you know, that's that's the downside of being outdoors. When it rains, obviously, it's a bit of an issue as well. You know, so, you know, so that's the downside."*<br><br>*"Shade is a big thing for me because I just the sun, I just find it so hot you know the Australian sun is very severe. And I'm quite pale so."*<br><br>*"There is no park lights or anything so sometimes it can be quite dark in the winter when you get there. The suns up by the time you leave which is quite nice but there's no shelter or whatever if it was raining and things like that."*<br><br>*"There's no toilets or anything there so yeah that could probably be turn someone off maybe I don't know."*

			Women's personal situation e.g. finances, childcare duties	*"It's the kids, and because I believe once COVID had come into play in the reopen I think I've got told that the kids couldn't come because of the with, with the COVID restrictions or something like that so that was a barrier and then for me I just was, and then it just becomes a week you miss, two weeks you miss, three weeks you miss and it's like, oh my goodness it's now a few weeks, you know. I'm pretty sure kids can come back again now but they have to make, make sure that they able to be self-sufficient."*<br><br>*"Instead of me going back home and being all miserable and like oh I gotta clean the house or this is never ending and it's that mental thing because then it's like you go back to home and it's like Groundhog Day, where when you go to these, these type of fitness programs you meet people and then you're like oh let's go for coffee and let's go, you know, do this or that, and you find out that people are on the same journey as you or similar or they've experienced that journey. And so they help in a way."*<br><br>*"I have a mix. It's a mixed. Some people are like yeah we wanna check it out and then some are like oh we've got kids and what happens then and it's like no no you can bring the kids. So they're a bit hesitant you know because they've never done something like that, like, and it's like, no, it's great. But then there's some people... I've actually had one friend go, 'if it's free then, it must be not like you know, like it's just not real exercise'. I don't know how she explained it and I just was like, 'what!?'"*<br><br>*"I used to be involved in a gym but I, you know, I mean I'm on limited income so you know some gym memberships get a little bit expensive. This is free you know so I thought it was a good opportunity to at least try it out if you don't like it well you're not wasting any money."*<br><br>*"Last year, we had a fantastic opportunity where a lady's son who was probably about eight, he exercised with us, and the trainer was so beautiful, because children are very usually very confident. He said to the child hey, you can you can be my supervisor, you can make sure all the ladies are doing the exercise, and that it boosted the kids confidence so not just people without children, but people with children can feel comfortable there and the trainers is make you feel comfortable, and if the trainer makes you feel comfortable, everyone else begins to comfortable with everyone else."*<br><br>When asked why she joined LLGA, *"Truthfully, the fact that it was free. That was a big plus for me because, like I had stopped working and one of the reasons why I had, I had stopped working, is you know I needed to help my daughter, a little bit more with her child. So, I've found there's like people like me in those classes and so, you know, to now I have to really watch money so that you know I don't know if it's a good thing or not but certainly that was a big factor for me."*<br><br>*"Sometimes it's timing too and yeah because cause I look after mum so like, yeah, sometime. Yeah, just, I'm rushing doing school drop off and stuff like that."*<br><br>*"You can bring your kids along, and which is good because like, sometimes the trainers they step in and help to so I've seen that in, and a majority of kids, little ones, they're just and the mum's just bring whatever they need and they just sit there and do their own little thing in the corner."*<br><br>*"I think, well you know I've basically worked my whole life, so I guess it was time and then you know you're tired, you're raising a family. You're working. So I guess that's been a factor. And sometimes you just don't feel, you can't be bothered. You just don't feel motivated. I've been around that loop many times."*
			Resources	When asked what makes a good trainer, *"Good personality. Like fun and outgoing and motivated. I think all the trainers are like, they've got a good personality like they're nice people."*

				"They were all professional instructors as well and the instructor, the field instructors I had were fabulous actually."<br><br>*"I am sun, sun conscious, may be a Cancer Council sun cream maybe 50 or 30 plus."*<br><br>*"Maybe in these times because of COVID maybe some hand sanitiser, wipes you know whatever is convenient."*<br><br>*"All the equipment's there too, you know like you've got your weight's, you got your dumbbells, you've got the resistance bands, you don't have to go out and buy anything, sort of for home so it's all there."*<br><br>*"They don't have a creche, which is understandable like when it's something that's, you know free, of course, you're not going expect a creche and things like that."* When asked if she would use a creche if LLGA had the resources, *"Oh for sure. Yes 100%. I'd go twice a day."*<br><br>*"I thought it would be awesome while the parents like the mums cause mainly it's mainly is mums, or retirees, but the mums with the kids, if they have another program for the kids while the mums are doing that the exercising the kids can too."*<br><br>*"With the St Mary's as well the time also. Sometimes the timing because I think it's 9:30 they do and so, like, 9:30 is great like even if it's earlier, that's fine. But also, it would be nice for an afternoon session."*<br><br>*"I think, having more more options. So for example like having a Zumba class or having what is it called... I don't know like high intensity type thing like I know that they've got boxing and things like that but I'm not a, it's not, it doesn't fancy me."*
	Social	Social influences	Community social support, influence and interactions	*"You become friends with people and you you know you tend to after training, you get to sit for five minutes and just chat and it might turn into, 'hey, what do you doing? Wanna want to meet up at Woolworths and continue shopping together and get groceries?'"*<br><br>*"The social and if you're struggling they're like, they tend to, we tend to help each other it's like oh you can do it. Come on, you know, Good on you. Good work. And it's like, you see each other you know oh wow you've lost weight, oh wow you look good, you know."*<br><br>"It's quite a friendly class. I think, I think also for some people's mental health is good. Yeah especially any types of people who are not having a lot of social contact and and that would include me because you know when I'm not working and I'm and especially with how things are been with us not being able to travel and being in contact with other people and it's friendly you can sort of... we we tend to joke around a little bit."<br><br>*"I'm better exercising with people, you know, I do a little bit by myself but I'm not as disciplined by myself so it's better if I'm with other people."*<br><br>*"I'd sort of told a couple of my other friends about it so like the three of us sort of like, you know, went together that first week."*<br><br>*"I'm better if there's a group to go to and I work with a group really I'm a bit more disciplined."*

				"Plus I've had good role models. My parents were both very very active. They were you know like my dad walked everywhere and swam every day of the week when he retired. My mum's always walked. She used to go to exercise classes and hydrotherapy and that so I've had good role models as well."<br><br>*"I'll post it on my pages like Instagram then it goes over to Facebook."*<br><br>*"If the places have like trainers from my community too like that's another thing too is that a lot of these people they're from my community. Someone knows them and then families and that go and support each other, where I reckon if we had something like that too like, one of the trainers and yeah that will and then, I reckon that will like will get out to my community and all different ethnics too."*<br><br>*"It's quite social in the sense that you know regulars and you get to know them and have a bit of a chat and some, some occasionally might go for a coffee with somebody. So, I think it is very important. Has been for me, cause with retiring you think, oh, what am I gonna do now and well will I make any friends or, you know, like, it's a big scary thing giving up full time work, you know all those years to yeah stop all of a sudden. So yeah it's been good to know that you know there's friendly people out there and even if it just that 45 minutes, an hour just to have a chat with somebody."*<br><br>*"It does have that role of creating a more of a community."*<br><br>*"The trainers encourage you, so they'll be like, "see you next session", and everyone will go, "we'll see you next time." So, because you create friendships, you kind of want to show up more because it's, it's like the people there become like a family. You can't wait to see them again. You can't wait to have that safe space and that support, and everyone's reaching their own goal, but together they're also reaching the goal as well which is, building a better version of themselves, mentally and physically, and also being supported."*
			Social status, pressures and comparisons	When speaking of gyms, *"it can be a bit intimidating with all the machines and the people with big muscles and so forth."*<br><br>*"You don't have to wear, it's comfortable clothes you don't have to wear any particular like gym clothes or anything like that, so I think it's, you know, quite easy really."*<br><br>*"I would feel quite intimidated walking into a gym class with a lot of young people. I just wouldn't feel comfortable."*<br><br>*"I suppose everyone's different, but I didn't mind like, because I know a lot, some ladies, they didn't like to train with men, but I didn't mind like we're all there for a purpose."*<br><br>When asked about their family and friends' response to her attending these classes, *"Well, some people some people like at first, some of my family said, 'oh, you know, you're going there to St Mary's in the park, oh my god.' They're more judgmental than I am. I'm pretty you know like, it's okay. All right, people are just people."*<br><br>*"I know this is gonna sound really bizarre, but actually finding clothing that's comfortable to wear. I know this one might sound funny, because, because I work shift work and all that I normally can't go shopping and all I've got is generally my work clothes."*

				"My mental health didn't feel comfortable being in a gym because of my social status, not having much money, and so, when I walked past live life get active I was like, they told me it was free they told me what they run, and I was just intrigued, because I since childhood has been a community person."<br><br>*"Looking at gyms, all I noticed was, it was like a competition with everyone else, instead of people competing with it own self, looking back on who they were and how they can be a better version of themselves. At the gym I noticed an occurrence of every time I wanted to go there, I felt like I had to wear expensive clothing because me in childhood was aware that when you go to the gym you wear what you're comfortable in when you exercise. In this generation and this era I feel it has become very competitive and expensive."*<br><br>*"It's a struggle to be able to fit a certain image to be able to attend the gym."*<br><br>*"It begins with the trainers. If you have a trainer that that's approachable that's comfortable, that has good personality, that that dresses comfortable, rather than to meet, meet a certain social status in the society."*
**Motivation**	Reflective	Beliefs about capabilities	Confidence and comfort level	*"I was attending some other classes but they were for I joined them for, they were for over 50s, or over 55s or something and because I I wasn't all that confident about joining a regular open aged class."*<br><br>*"I did feel stronger and my joints are a lot better um and yeah I feel more confident, stronger definitely, able to move around better. More confidence in my strength and yeah in that I'll be able to continue function without without help."*<br><br>*"Oh no, gosh no it's not competitive at all, you know, and I mean I don't think that that's not the idea of these at all. I think the idea of this group is just to improve, you know like start somewhere and just keep on improving."*<br><br>*"When you go to any first time thing, you get really nervous but yeah because I didn't really know what to expect but I got there and I seen that there were older people older than me."*
		Beliefs about consequences	Deterioration of health and ageing	*The participant speaks about the consequences of inactivity and how it makes her feel, "Down very down and that's how I feel at the moment because I haven't gone for a few weeks and so for me it does affect you know mentally and it's like that, I want to rip that band aid and start going back but I know because school holidays is coming up and it's that cringe-ness of all my goodness, I have to drag along all four kids."*<br><br>*"I think it's just that well it's mostly health for me because I think, you know, like I've been nursing so I I've seen a lot of people, you know in hospital, it doesn't take long for people to decondition and I think if you sit around and don't do anything, you lose, you lose a lot of strength, you lose a lot of muscle strength, you know, eventually problems with balance and things like that so I just don't want to end up like that. I want to try and keep as active as I can for as long as I can."*<br><br>*"Well it's very important role for me physical activity and personally, I guess I believe that the older you get, the more important it becomes. You know, for health and fitness reasons and for general well-being."*<br><br>*"When I'm not as active I feel very sluggish. Yeah, and maybe even a bit down, you know, I just don't feel as as good in myself, kind of thing. And I feel like just grumpy when I'm not active."*

				"What it means to me is, you know, if you, if you don't use it, you lose it so it's about you know health, staying flexible, yeah, getting less aches and pains, you know, sort of wards off illness and helps you to lose weight, yeah and also good for the mood as well for mental health."
		Intentions	Weight loss	*"Lose weight and just with it was just going through the breakdown of the marriage and things like that so I wanted to keep myself occupied and busy."*<br><br>*"Weight is like a probably a bit of an issue. I don't want to sort of get overweight, but it's more the health."*<br><br>*"I was doing the weight loss, but now I'm more, because I like food. So I just, just more workout to enjoy my food. Yeah. I don't know but I think the older you get, the harder it is to try and lose so I might as well just enjoy what I'm doing and enjoy eating food."*<br><br>*"I feel like my focuses is being like fit and healthy and strong. So, like I don't even weigh myself, and you know I'll put it out there I'm not really very that overweight, or I don't think I'm overweight but, and so that's good for me but, you know, I don't want to be weighing myself all the time just to put that in if you know what I mean because I don't want to become obsessed with that if you know what I mean."*<br><br>*"I have health conditions and mental health conditions so finances are very tight and have been for quite a few years and part of my health conditions was to lose some weight."*<br><br>*"I want to have muscles, I want to… I want to look good, I suppose, yeah I don't, I don't want… I have put on kilos now that I need to lose but I just prefer to keep myself in shape."*
			Goals	*"I'm pretty much retired and I made a priority to increase my health cause I sort of wasn't feeling as healthy as I might be and yeah it was it was more difficult to get to before when I was I was working and all of that."*<br><br>*"Mainly health reasons, really. I've got a little bit of osteoarthritis in my back and my hips so I always do a bit of stretching anyway because I find that does help, but I think it's mostly health. I mean, I don't want to sort of get overweight, but it's more the health and so I just want to keep muscle strength, I'm aware of osteoporosis, my blood levels I'm sort of like creeping up a little bit you know like to pre diabetic levels and that so I just don't want to get that out of the hand."*<br><br>*"I think having a time is a good motivator because I think if you just try and fit in exercise anytime, often you don't end up doing it. I think sometimes like if you, you look out and you find oh it's a bit windy today or a bit hot today you know, you think nup won't go, or, you know, it's time if you've got you know things you've, you've got to get done, or you think, oh no I won't go today I'll go next time you know that can get into a little bit of a habit really. I think, you know, some people seem to be really motivated. Some people go five days a week, I couldn't do that. I couldn't do it five days a week you know it just wouldn't. That wouldn't suit me at all. So some people are really motivated. For me, I think twice a week's okay."*<br><br>*"I feel like my focuses is being like fit and healthy and strong. So, like I don't even weigh myself, and you know I'll put it out there I'm not really very that overweight, or I don't think I'm overweight but, and so that's good for me but, you know, I don't want to be weighing myself all the time just to put that in if you know what I mean because I don't want to become obsessed with that if you know what I mean."*

				"As far as exercise and the health and that you know you can't take the horse to water, the horse has to take itself sort of thing so I tend not too overly stress out whether people do take upon it or not."
			Independence in older age	*"I was getting a little bit, some of my joints are getting a bit stiff and I sort of felt I was losing flexibility and especially my knees and ankles were... I was at one stage having a bit of trouble on stairs and that worried me and sort of losing mobility because yeah, mostly because I'm a single person and I've got no one to push a wheelchair so yeah and I don't drive either so I'm always on enough trains and walking yeah so I really don't want to lose mobility I think that that's a big a big issue."*<br><br>*"That's a big issue yeah to maintain strength and mobility and yeah not accepting, I'm not accepting getting older and weaker. I want to keep myself together for a while."*<br><br>*"I did feel stronger and my joints are a lot better and I feel more confident, stronger definitely, able to move around better."*
	Automatic	Reinforcement emotions	Rewards and incentives	*"What I think would be great is having like a challenge. 60 Day Challenge or, you know, how some places, some personal places, personal training places they do 60 day training or no what is it? 60 day weight loss camp, bootcamp or something like that? Then people can come and go 60 days or 30 days, whatever it is, and then people are like okay we're coming for a certain period of time, and we're able to, it's like having the goal in that timeframe, and you're able to smash it and even if there's a reward at the end of it where it could be just movie tickets for two people. It doesn't have to be anything like for me I'd be happy with the movie ticket."*<br><br>*"Just every now and then, I mean not all the time maybe having some sort of competition or something. Sign up a friend or something, win something nice-ish."*<br><br>*"That's something I've really embraced and has encouraged me because me seeing how many sessions I've attended, I'm able to go wow, I've attended that many, I want to attend more because, oh wow, this person or he has reached their milestone of 50 sessions. I want to go because I want to reach the 50."*<br><br>*"So every, every goal you reach is celebrated. You can also recognise that, for example myself, I was also able to notice okay, even though my weight has gone up, my waistline has shrunk so I know that I'm gaining muscle. And so it's also, they've got educational tools for you to recognise your own milestones and that, that is, that encourages me so much more because it's like, it's like, it boosts my dopamine levels pretty much so like completing a task so every time I complete a session, it's like completing a task and I feel so good about myself I feel proud of myself and I can and with being able to reflect on how far you've come through your profile, you're able to, I guess, get this confidence boost about you, you're able to validate and feel proud of yourself, so I can sit here and go, 'wow, I've done this myself.'"*
			Enjoyment	*"I like greenery. So you go on a walk, I want to walk where there's trees and greenery and birds, and all that sort of stuff."*<br><br>*"I find it much more motivating in a class. I do like going to a class. Personally it's not so much the social thing really, but yeah I do I do just love the structure of the class. I like the instructor up there and it is a bit social, that's kind of a bonus."*<br><br>*"It's good and if people and I noticed everybody is pretty friendly, too. So, you know, I feel like maybe I don't need so much the social aspect but other people may. So I think that's really good because, and you are with like-minded people who probably value exercise. So I think that's that's another nice thing."*

				"I cannot get motivated here in the house. But when… the thing is when the normal camps are running, I still do attend the online at home, I managed to do them at home I still had motivation, but when the actual camps close, for some reason I try and do them at home and I've got… the motivation for some reason isn't there."<br><br>*"I enjoy it, I love it, I love going to it. I've liked all the trainers too. I thought they've all been really good. They've all given me motivation to train and I like the fact that they take photos of me too."*<br><br>*"I love it. It's one of the things that I like doing, I like, I like moving all the time."*
